# The effect of a fibrin sealant on knee function after total knee replacement surgery. Results from the FIRST trial. A multicenter randomized controlled trial

**DOI:** 10.1371/journal.pone.0200804

**Published:** 2018-07-25

**Authors:** Wiebe C. Verra, Joost A. van Hilten, Áine Honohan, Erik W. van Zwet, Johanna G. van der Bom, Rob G. H. H. Nelissen, on behalf of the FIRST-research group

**Affiliations:** 1 Department of Orthopedic Surgery, Leiden University Medical Center, Leiden, the Netherlands; 2 Center for Clinical Transfusion Research, Sanquin Research, Leiden, the Netherlands; 3 Department of Clinical Epidemiology, Leiden University Medical Center, Leiden, the Netherlands; 4 Department of Statistics and Bioinformatics, Leiden University Medical Center, Leiden, the Netherlands; Harvard Medical School, UNITED STATES

## Abstract

**Background:**

Total knee replacement (TKR) is increasingly performed in short term hospital stay, making same day mobilization an important issue is after surgery. This implies little joint effusion by reducing intra-articular blood loss, which will enhance knee range of motion. The application of a topical fibrin sealant on the intraoperative bare bone and synovial tissue may contribute to better early full mobilization and thus improved functional outcomes. Since ambulation with a fully extended knee is less strenuous, we hypothesized that patients who received fibrin sealant would demonstrate improved early knee extension after six weeks compared to patients who received standard care.

**Methods:**

A multicenter randomized controlled trial in a consecutive series of osteoarthritis patients scheduled for TKR surgery. Participants were randomized to receive fibrin sealant or not before closing the knee joint capsule. Primary outcome was change in knee extension angle(°) at short term (2 weeks) follow-up (cExt). Secondary outcomes were 6-week extension angle, knee flexion angle, hemoglobin loss, blood transfusion rates, complication rates, the Knee Society Score, and the KOOS and EQ5D questionnaires.

**Results:**

When data on primary outcome became available from 250 patients, an interim analysis was performed by an independent Data Safety Monitoring Board for safety and effectivity assessment. This analysis showed that sufficient patients were included to detect a cExt of 10° between both groups. Inclusion was stopped however, all in the meantime included patients were treated according to their randomization. A total of 466 were available for analysis. Both groups were comparable in terms of baseline characteristics. The estimated mean cExt difference was 0.2° (95%CI -0.5 to 0.9). No differences in secondary outcomes were found.

**Conclusions:**

No beneficial effects or side effects were found of a topically applied fibrin sealant during TKR surgery. These results discourage the clinical use of a fibrin sealant in TKR.

**Trial registration:**

Dutch Trial Register, NTR2500.

## Introduction

The frequency of total knee replacement (TKR) procedures performed for the treatment of knee osteoarthritis will increase in the coming years due to an aging population [1]. In the Netherlands the number of TKR increased by almost 25% between 2010 and 2015 to over 26.000 TKR annually (www.lroi.nl). TKR is also increasingly performed in two-day or even one-day surgery, necessitating the need for immediate postoperative full ambulation and range of motion exercises. Since the latter is restricted by intra-articular blood loss, means to control this loss are important for rapid patient recovery. The mobilization and weight bearing are less strenuous if full extension of the knee is present. On a more holistic patient level, these issues have also been shown to be related to patient blood management [2–7]. Earlier, our group demonstrated an overall average of 650–700 mL of total (visible and non-visible) blood loss after TKR [4]. Reducing this blood loss will most likely benefit the TKR patient.

Theoretically, a fibrin sealant has the ability to reduce bleeding of surgically injured bone and synovial tissue by forming a sealing layer [6]. Several randomized studies report on the effect of fibrin sealant in reducing blood loss (i.e. hemoglobin level) and/or transfusion rates after TKR [8–14]. Since the introduction of modern transfusion trigger protocols transfusion rates have decreased tremendously and reducing transfusion frequency has therefore become a less relevant outcome after TKR. Outcome measures such as improvement of function and mobility are increasingly considered important, improving patient independence and satisfaction.

We designed a randomized controlled clinical trial to assess the effect of a topical applied allogeneic single donor fibrin sealant on functional knee recovery after TKR surgery. We hypothesized that patients who received this topical fibrin sealant intraoperatively would demonstrate improved clinical favourable early knee extension (primary endpoint) compared to patients who received standard care.

## Methods

We conducted a single-blinded, multicenter randomized controlled trial at six orthopedic centers in the Netherlands. The study protocol was approved by the central medical ethics committee of the Leiden University Medical Center (P10.115) and registered at the Dutch Trial Registry (NTR2500). Local medical ethics committees approved the study protocol in all participating centers (these committees were METC Brabant, METC Zuidwest Holland and METC Noord Holland). A study independent monitor visited one of the centers to monitor legal-and protocol compliance.

### Patients

Patients elected to undergo primary TKR between January 2011 and February 2013 for the treatment of primary osteoarthritis or rheumatoid arthritis were eligible to be included in the study. Exclusion criteria were age under eighteen years, ASA score >III, any congenital or acquired coagulation disorders (hemophilia or von Willebrand disease, INR >2.0, liver failure), no knowledge of the Dutch language, and unwillingness to participate. All patients provided written informed consent before inclusion.

Patients were randomized to receive either intra-articular topical Cryoseal^™^ fibrin sealant (CS) or standard care without an intra-articular hemostat [15]. A method of computer generated per-center randomization using permutated blocks with randomly differing block-sizes was used (ProMISe^™^ software; Leiden University Medical Center). Patients, all staff involved in data collection and data analysis and all authors were unaware of the treatment allocation.

### Investigational product

Cryoseal^™^ fibrin sealant (CS) is produced by Sanquin, The Netherlands [15]. CS is derived from one unit of fresh frozen plasma donated by a single donor. One unit of single-donor quarantined plasma yields between 10–15 mL CS from which two (5 mL+3–5 mL) syringes were transported in a sealed bag. A fibrin sealant in general is composed of two main components, fibrinogen and thrombin that, when mixed together at 37°C results in a fibrin molecule clot.

### Protocol of surgery

All patients were operated according to the study protocol and the manual of the TKR used. Type of anesthesia was not standardized. Tourniquet use during surgery was allowed; however, during the procedure the tourniquet was deflated in order to surgically coagulate injured tissue with electrocautery. Timing of deflation of the tourniquet was left to the orthopedic surgeons’ preference. All participating hospitals were free to choose their own preferred brand and type of TKR implant. Cementation was left to the centers preference. We hypothesized that the use of drainage systems may interact with the effect of the CS. Orthopedic centers were therefore requested to perform the procedure either with or without vacuum drainage for all TKR procedures at that center.

For each randomized patient a cooling box was delivered to the operating room containing cooling elements and either CS or no CS. Before application the frozen CS was thawed at 40°C for at least twenty minutes. The surgeon and scrub nurse were only informed about the content of the box immediately before application. Patients assigned to the CS group were treated with a maximum of 10 mL CS divided over two separate syringes, one with 5 mL and one with the remaining 3–5 mL. The use of at least 5 mL CS was mandatory. The CS was topically applied after placement of the implant on intra-articular tissues and bare bone surfaces. CS was applied with the use of a spray tip mounted on the syringe. The remaining 3–5 ml CS was used at the discretion of the surgeon. The knee was closed routinely. All unused CS and empty syringes were returned to the local blood transfusion department where the amount of CS applied to each patient was recorded. Standard care was considered TKR according to this protocol without the use of CS.

After surgery all patients received a low molecular weight heparin thrombosis prophylaxis during six weeks. All patients followed a regimen of full weight-bearing physical therapy.

### Transfusion policy

Decisions regarding perioperative blood transfusion were made by the attending anesthesiologist and/or orthopedic surgeon, similar guidelines were in place in all participating hospitals. The transfusion protocol is presented in the Supporting Information (S1 File. **Transfusion protocol**).

### Data collection

Data were transcribed onto Case Report Forms (CRFs) by research nurses who were unaware of the randomization result. All written data were transferred from the CRF to the secure web-based data management system (ProMISe^™^).

### Outcomes

Primary outcome was the change in knee extension (cExt) angle (°) at short term follow-up (i.e. after two weeks) compared to the pre-operative knee extension.

Secondary outcomes were the 6-week cExt, the knee flexion, perioperative blood (hemoglobin) loss, transfusion rates, postoperative pain, complications (superficial and deep infection, hematoma, and systemic complications), and total duration of hospital stay. Furthermore, the Knee Society score and validated patient reported outcome scores; the Dutch versions of the Knee Injury and Osteoarthritis Outcome Score (KOOS) [16] and the EQ5-D (descriptive system and EQ quality of life as VAS score) [17] were recorded. Outcomes were recorded at baseline and 2-6-and 52 weeks after surgery.

### Sample size

A sample size calculation was performed for our primary outcome which is cExt two weeks after surgery. A difference between study arms of 10° was expected and was also considered clinically relevant. Because of scarcity of data to base our calculations on, based on the date from a trial registered on clinicaltrials.gov (NCT00492219) a standard deviation of 35 degrees was assumed. The sample size needed to detect a difference of 10° with a t test assuming equal standard deviation in both groups of 35 is 259 per group (using the O’Brien-Fleming rule for one interim analysis). Because of the scarcity of data during development of the study protocol a re-estimation of the sample size was specified in the protocol after the first 250 inclusions were completed.

### Interim analysis

According to the protocol a single interim analysis was conducted by an independent Data Safety Monitoring Board (DSMB) when 2-week follow-up data were available from 250 patients (because of overshoot this turned out to be N = 262 included in interim analysis). The interim analysis was intended as both a safety assessment and superiority analysis as well as used to re-estimate the sample size.

Ultimately an interim analysis of the first 262 evaluable patients was performed. All (serious) adverse events were recorded. The DSMB judged whether an adverse event was possibly related to treatment with CS. The DSMB was blinded to group allocation when assessing the data. The standard deviation of cExt between baseline and 2 weeks was 7.7 according to the interim analysis. It was concluded that in the study protocol the standard deviation of the primary outcome was over-estimated. According to this new sample size calculation there was already enough power to stop inclusion. However, because the protocol stated at least 400 patients were to be included, it was decided to continue until this amount was reached. Ultimately more than 400 patients were included because of overshoot of inclusion by the participating study centers.

### Statistics

Descriptive statistics are reported as number and percentage for categorical variables. Normally distributed continuous variables are reported as mean and standard deviation and non-normally distributed continuous variables as median and inter-quartile range.

#### Primary outcome

A repeated measure linear mixed model was used to assess the difference in cExt between patients randomized for Standard Care and CryoSeal fibrin sealant, adjusting for pre-operative knee extension angles (crude model).

The model was adjusted for any misbalance in baseline characteristics between the randomized groups (Model 1). To investigate whether the CS effect was modified by the use of a drain, drain use and the interaction between drain use and CS vs standard care was added to the model (Model 2).

#### Secondary outcomes

For the secondary outcome change in knee extension after 6-week and for change in knee flexion, the same repeated measurement analysis of covariance was performed as for the primary outcome adjusting for preoperative knee flexion. EQ5D and VAS were compared by mean and interquartile range for both randomization groups pre-operatively and after six weeks of follow-up.

#### Post-Hoc

In a post-hoc analysis patients with a marked preoperative knee extension of ≤ -15° (meaning a knee flexion contracture of less than 15°) were selected to investigate whether there was a difference in cExt between Standard Care and CS in this patient group. An interaction with drain usage was also investigated between these groups.

Analyses were carried out according to the intension-to-treat (ITT) principle. Difference in estimated mean differences between CS and Standard Care arms and their 95% confidence intervals were computed with the Standard care arm as a reference group.

Statistical analysis was performed with computer software (SPSS 20.0 for Windows, SPSS Chicago, IL.). Statistical tests were two sided, a p-value of <0.05 was considered statistical significant.

## Results

**Fig 1:**
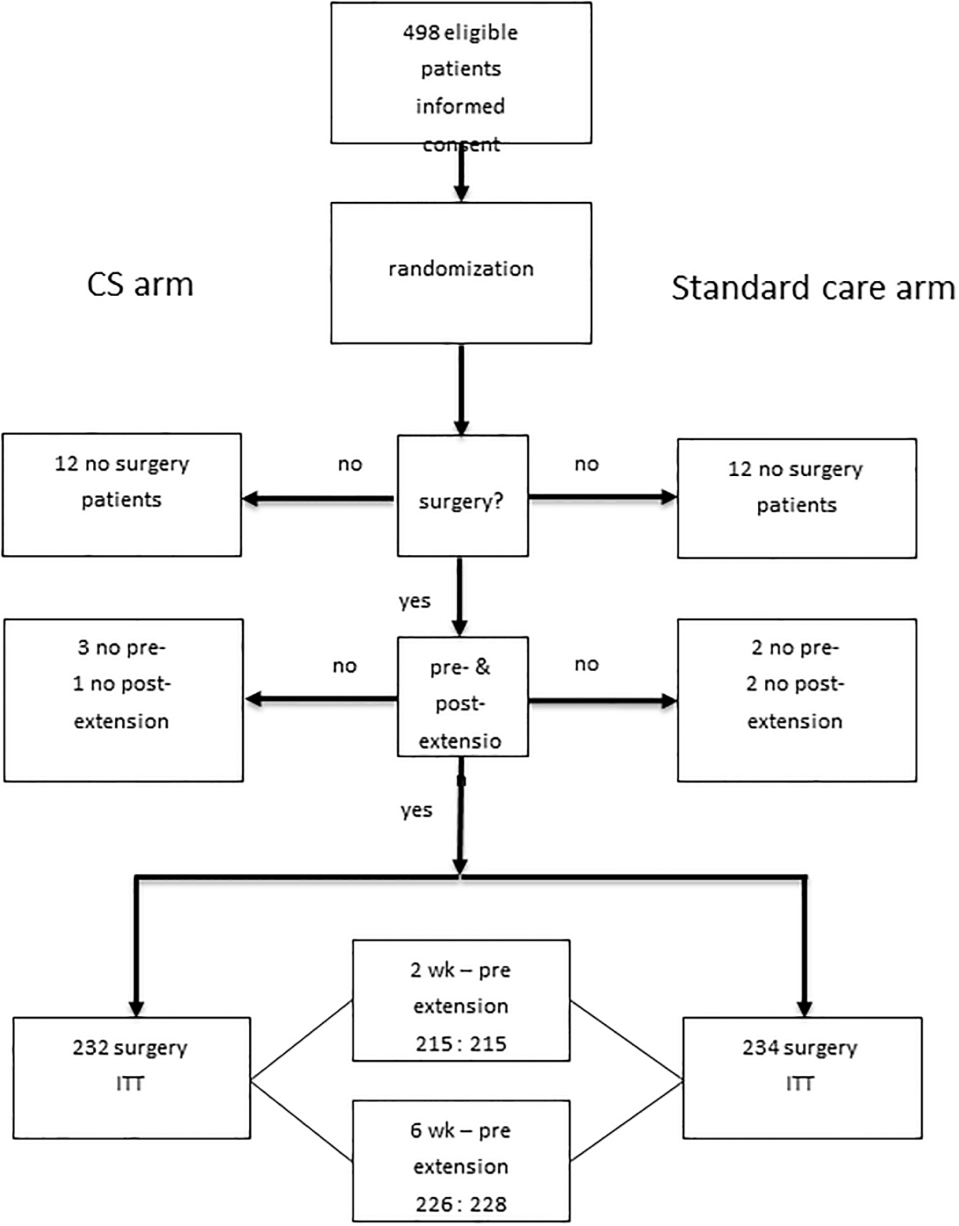
CONSORT Flow Diagram of patient inclusion.

A total of 498 patients were randomized between January 2011 and February 2013. From these patients a total of twenty-four (twelve patients in each study arm) ultimately did not undergo TKR surgery or withdrew their informed consent (IC). A further four eligible patients (3 in CS arm and 1 in control arm) gave IC twice and were included by randomization for a second TKR at least three months later on the contralateral side. Eight patients who underwent TKR were excluded for analysis due to the missing cExt data pre-or postoperatively. A total of 466 patients were available for analysis; 232 in the CS arm and 234 in the control arm (Fig 1). Due to random logistical reasons with the different clinics no exact total of patients who were eligible can be presented.

### Patient characteristics

Table 1 shows pre-and peri-operative characteristics of randomized patients. There was no difference in baseline patient characteristics except a higher incidence of diabetes mellitus in the control arm. One center used uncemented TKR (15%), all other centers used cemented TKR systems.

**Table 1:** Characteristics of participants.

	Standard Care	CryoSeal
**Baseline variables**		
Number of patients	234	232
Females N (%)	152 (65)	148 (64)
Age years(SD)	68 (9)	68 (10)
Age years range	41–87	41–88
Body mass index (kg/m^2^) (SD)	29 (5)	29 (5)
***ASA score*** *N (%)*		
I	44 (19)	32 (14)
II or III	182 (78)	185 (80)
***Associated co-morbidity*** *N (%)*		
Diabetes Mellitus	47 (20)	31 (13)
***Type OA*** *N (%)*		
Primary OA	215 (92)	203 (88)
**Pre-operative**		
Hemoglobin (g/dL) mean (SD)	13.8 (1.3)	13.7 (1.4)
Pain score (0–10) median (IQR)	7 (5 to 8)	7 (5 to 8)
Knee extension angle (°) median (IQR)	-2.5 (0 to -5)	-5.0 (0 to -10)
Pre-operative extension deficit ≤15° N (%)	26 (11)	37 (16)
Flexion angle (°) median (IQR)	110 (100 to 120)	110 (100 to 120)
**Peri-operative**		
Surgical time minutes (IQR)	75 (60 to 100)	76 (62 to 97)
Length of hospital stay days (IQR)	4 (3–4)	4 (3–4)
Drain system used N (%)	87 (38)	79 (34)
Drain production mL (IQR)	477 (312 to 730)	550 (325 to 760)
RBC transfusions N (%)	7 (3.0)	6 (2.6)

Abbreviations: N, number; SD, standard deviation; IQR, interquartile range; ASA, American Society of Anaesthesiologists score; OA, osteoarthritis; RBC, Red Blood Cells

### Primary outcome

The results of the intention-to-treat (ITT)-analysis mean change in postoperative knee extension (cExt) for patients randomized for standard care and CS after 2 weeks are shown in Table 2.

**Table 2:** Primary outcome, cExt, two weeks after TKR.

		Mean cExt (95%-CI)
Model 1		
(adjusted for DM)	Standard care	1.2 (0.5 to 1.8)
	CS fibrin	1.0 (0.3 to 1.6)
Model 2 (adjusted for drain)		
Drain +	Standard care	0.9 (0.1 to 1.7)
	CS fibrin	1.7 (0.7 to 2.6)
Drain -	Standard care	1.3 (0.6 to 2.1)
	CS fibrin	0.6 (-0.2 to 1.3)

cExt: mean change in extension, TKR: total knee replacement, 95%-CI: 95% confidence interval, DM: diabetes mellitus, CS: CryoSeal

The overall estimated mean cExt at short term follow-up after TKR was comparable between Cryoseal (CS 2.0° (95%CI 1.6° to 2.5°) and standard care 1.8° (95% CI 1.4° to 2.3°); mean difference of 0.18° (95%CI -0.85 to 0.49).

Both arms were comparable after adjusting for misbalance in randomization for diabetes (model 1), furthermore there was no interaction between drain usage and CS (model 2).

### Secondary outcomes

Both study arms showed equal improvement in cExt at 6 weeks compared to 2 weeks (S1 Table). There was no noteworthy difference in change in short term knee flexion in CS patients compared to standard care patients (S2 Table). Furthermore, there was no difference in length of hospital stay between both groups (median 4 days, IQR 3–4 days for both groups). The Knee Society and Knee Functional score significantly improved after surgery, however comparing the scores between both treatment groups did not yield a difference (S3 Table). The EQ5D VAS was also similar for patients treated with CS and with standard care. All subscales of the KOOS improved after the surgery however there were no differences between the treatment groups (S1 Fig).

### Complications

Postoperative (serious) adverse events were scored up to one year postoperatively. Table 3 shows the complications per treatment arm. Complication rates were low and similar for the two intervention arms.

**Table 3:** Complications.

	Standard Care	CryoSeal
Wound infection	5 (2.1)	8 (3.4)
Deep infection	1 (0.4)	3 (1.3)
Manipulation knee (OR)	3 (1.3)	0 (0)
Manipulation knee (ward)	3 (1.3)	1 (0.4)
Knee hematoma	1 (0.4)	4 (1.7)
Pneumonia	1 (0.4)	2 (0.9)
Urinary tract infection	4 (1.7)	3 (1.3)
Admission ICU	1 (0.4)	2 (0.9)
Cardial events	10 (4.2)	6 (2.6)
Respiratory events	4 (1.7)	1 (0.4)

Complications are reported as number (% of treatment group)

### Post-hoc analyses

Only 63 (14%) patients showed a pre-operative knee extension i.e. ≤ -15°, 37 (16%)in the CS group and 26 (11%) in the standard care group. While both treatment groups showed a clinically relevant early cExt post-operative (standard care 16° (95% CI 14 to 17), CS 15° (95% CI 14 to 16)) there was no clinically relevant difference in cExt between treatment groups. Furthermore, use of the drain showed no additional benefit in either group (S4 Table).

## Discussion

Topical application of CS did not improve postoperative knee extension at short-term (i.e. 2 week) follow-up after TKR compared to standard care.

For this study a difference in extension angle of 10° improvement or more was defined as clinically relevant [18]. However, this pre-defined clinical relevant knee extension appeared not feasible as in our cohort the median pre-op extension deficit was only 5° (IQR 0 to 10). Nonetheless the study results also accentuate that despite extensive surgery to a knee joint (i.e. TKR) which create large bleeding surfaces, the intra-articular blood loss does not seem to interfere with short-term range of motion.

Two meta-analyses studied the effect of fibrin sealant in TKR surgery, both showing a reduction of postoperative blood loss in the fibrin sealant group with a subsequent decrease in postoperative drainage and red blood cell transfusion rates [2,14]. Both meta-analyses found no difference in complication rate between fibrin sealant and control groups. In contrast to our study, Wang et al. showed a significantly improved overall mean range of motion (i.e. flexion to extension) of 16° in patients (N = 144) treated with a fibrin sealant compared to those who were not treated with fibrin sealant [14]. However, this pooled mean was based on a small number of patients from only 2 studies with significant heterogeneity and no conclusion could be drawn.

Preventing blood loss peri-operatively may include numerous strategies. Intra-operative strategies could include administration of pharmacological agents, i.e. tranexamic acid application, but also the use of topical hemostats on the surgical field (i.e. fibrin sealant) [3,6].

Since generic measures for patient blood management have reduced blood transfusion considerably, focus within blood management has also shifted towards improvement of quality of life and functionality of the patient [4]. We therefore addressed the surgical bleeding area, since this has impact on early ambulation as well as mobility of the surgically treated joint.

An analysis of functional outcome as primary outcome (i.e. knee extension) has not been investigated in the context of patient blood management. Knee extension deficit was used as a primary outcome since ambulating with a flexed knee is more strenuous for the patient with subsequent more energy consumption in the postoperative period. A recent small study (N = 48 knees) described the effect of fibrin sealant on blood loss and, for the first time in the literature, on early functional recovery defined by knee swelling, pain, range of motion and strength of knee extension [12]. Twenty-four patients receiving bilateral simultaneous TKA were analyzed with neither any benefit of fibrin sealant in this small patient sample. Another recent study evaluated the effect of topical application of fibrinogen in TKR in 200 patients, demonstrating no difference in terms of blood loss or transfusion frequency [19].

Small studies have been performed assessing the optimal dosage of the fibrin sealant application in TKR; 2 mL is considered too little while 5 mL was considered enough compared to 10 ml in a TKR study [20,21].

We studied a large sample of TKR patients in a prospective randomized controlled trial, with the passive extension deficit of the knee as functional endpoint. This is the first RCT with sufficient power to measure a putative effect of fibrin sealant on functional recovery of the knee. We advocate, since patient blood management is well implemented in current clinical practice in the Netherlands, that knee extension is a clinically more relevant outcome measure than transfusion rates and hemoglobin loss. Transfusion rates in the Netherlands were already low, being 11% in a total of 2.500 TKR and total hip replacement patients study on patient blood management in 2010 [4,22]. This has dropped even further to 4% in the current TKR study.

A limitation of our study is that we used standard care as control and also standard care with respect to the center’s preference to the use of a postoperative drain. It was considered that interference of the clinical practice during the study period (use or non-use of drain system) would cause a larger bias than just accept center wide use or non use of a drain. The study protocol allowed several factors to the preference per center (i.e. not individual preference). It should be noted that the sample size reassessment procedure we used did not follow rigor statistical reasoning to control for type I error. Another limitation is that measurements of knee angle were performed using goniometry, which is viewed by some to be imprecise. However, due to the large number of patients included, the randomized design and blinded analysis of the study data, even the small mean change in postoperative extension in outcomes could very well be clinically interpretable even more since inter-observer variability of range of motion measurements using a goniometer show good reliability in literature [23].

### Conclusion

This study demonstrated neither beneficial effects nor side effects of CryoSeal fibrin sealant on the functional postoperative recovery after total knee replacement surgery. There was no difference in change of knee extension after TKR between patient treated with fibrin sealant or with standard care. Also, no difference between these groups was found in change of other postoperative outcomes. These results discourage the clinical use of a fibrin sealant after TKR.

## Supporting information

S1 Table cExt after 2 and 6 weeks. (DOCX)

S2 Table Change in flexion angle. (DOCX)

S3 Table Knee Society scores. (DOCX)

S4 Table cExt after 2 and 6 weeks in patient with severe extension deficit pre-operatively. (DOCX)

S1 Fig KOOS subscales for both treatment groups. (DOCX)

S1 File Transfusion protocol. (DOCX)

S2 File Protocol FIRST study. (DOC)

S3 File CONSORT checklist FIRST study. (DOC)
