# Supplementary material for: The effect of a fibrin sealant on knee function after total knee replacement surgery. Results from the FIRST trial. A multicenter randomized controlled trial
Source: PLoS One. 2018 Jul 25;13(7):e0200804. doi: 10.1371/journal.pone.0200804 (PMC6059473; doi:10.1371/journal.pone.0200804)
Supplement: S1 Table — (DOCX) [file pone.0200804.s001.docx]

**S1 Table.
Mean change in knee extension compared to preoperative extension after 2 and 6 weeks in both drain and non-drain users**

|  |  | **Mean change extension angle (95% CI)** | | |
| --- | --- | --- | --- | --- |
|  |  | **Overall  (up to 6 weeks)** | **at 2 weeks** | **at 6 weeks** |
| Crude model |  |  |  |  |
|  | Standard Care | 2.0 (1.6 to 2.5) |  |  |
|  | CS fibrin | 1.8 (1.4 to 2.3) |  |  |
| Model 1  (adjusted for diabetes) |  |  |  |  |
|  | Standard Care | 1.7 (1.2 to 2.3) | 1.2 (0.5 to 1.8) | 2.3 (1.7 to 2.8) |
|  | CS fibrin | 1.5 (0.92 to 2.1) | 0.95 (0.3 to 1.6) | 2.1 (1.4 to 2.6) |
| Model 2  (usage of drain) |  |  |  |  |
| Drain + | Standard Care | 1.5 (0.7 to 2.3) | 0.9 (0.05 to 1.7) | 2.0 (1.2 to 2.8) |
|  | CS fibrin | 2.2 (1.3 to 3.1) | 1.7 (0.7 to 2.6) | 2.8 (1.9 to 3.6) |
| Drain - | Standard Care | 1.9 (1.2 to 2.6) | 1.3 (0.6to 2.1) | 2.4 (1.7 to 3.1) |
|  | CS fibrin | 1.1 (0.5 to 1.8) | 0.6 (-0.2 to 1.3) | 1.7 (1.0 to 2.4) |

**Results are expressed as estimated marginal mean cExt. angle with 95% confidence interval.
Overall (up to 6 weeks) and 2 and 6 week, change in knee extension angle after TKR for patients randomized for standard care or CryoSeal (CS) are shown as crude, adjusted (model 1) and interaction between the drain and randomized groups (Model 2). Crude model: is corrected for pre-op extension in accordance with the study protocol**
