# Supplementary material for: The effect of a fibrin sealant on knee function after total knee replacement surgery. Results from the FIRST trial. A multicenter randomized controlled trial
Source: PLoS One. 2018 Jul 25;13(7):e0200804. doi: 10.1371/journal.pone.0200804 (PMC6059473; doi:10.1371/journal.pone.0200804)
Supplement: S2 Table — (DOCX) [file pone.0200804.s002.docx]

**S2 Table.
Change in postoperative knee flexion compared to preoperative knee in both drain and non-drain users**

|  |  | **Mean change flexion angle  (95%CI)** |
| --- | --- | --- |
| Crude model | Standard Care | Ref. |
|  | CS fibrin | -0.7 (-2.6 to 1.1) |
| Model 1 | Standard Care | Ref. |
|  | CS fibrin | -0.61 (-2.5 to 1.3) |
| Model 2 |  |  |
| Drain + | Standard Care | Ref. |
|  | CS fibrin | 0.02 (-3.1 to 3.1) |
| Drain - | Standard Care | Ref. |
|  | CS fibrin | -0.9 (-3.2 to 1.4) |
