# Supplementary material for: The effect of a fibrin sealant on knee function after total knee replacement surgery. Results from the FIRST trial. A multicenter randomized controlled trial
Source: PLoS One. 2018 Jul 25;13(7):e0200804. doi: 10.1371/journal.pone.0200804 (PMC6059473; doi:10.1371/journal.pone.0200804)
Supplement: S3 Table — (DOCX) [file pone.0200804.s003.docx]

**S3 Table.**
**Knee Society Knee and Functional scores**

| **Knee Society Score** | **Pre-operative** | | **6 weeks** | | **1 year** | |
| --- | --- | --- | --- | --- | --- | --- |
|  | **Standard** | **CS** | **Standard** | **CS** | **Standard** | **CS** |
| Knee Score | 51 (17) | 51 (17) | 80 (17) | 78 (17) | 92 (10) | 90 (14) |
| Functional Score | 47 (21) | 46 (19) | 59 (23) | 56 (22) | 77 (23) | 76 (25) |

**Means (standard deviation). CS, CryoSeal fibrin sealant
For comparison reasons we added the 1 year results for the Knee Society Score.**
