# Supplementary material for: The effect of a fibrin sealant on knee function after total knee replacement surgery. Results from the FIRST trial. A multicenter randomized controlled trial
Source: PLoS One. 2018 Jul 25;13(7):e0200804. doi: 10.1371/journal.pone.0200804 (PMC6059473; doi:10.1371/journal.pone.0200804)
Supplement: S4 Table — (DOCX) [file pone.0200804.s004.docx]

**S4 Table.
Mean change in postoperative knee extension up to 6 weeks (overall) and at 2 and 6 weeks in TKR patients with pre-operative knee extension ≤ -15^o^ randomized for Standard Care and CS fibrin.**

|  |  | **mean change in extension angle (95% CI)** (N=63 patients with a pre-operative knee extension ≤-15) | | |
| --- | --- | --- | --- | --- |
|  |  | **Overall  (up to 6 weeks)** | **at 2 weeks** | **at 6 weeks** |
| Crude model | Standard Care | 16 (14 to 17) | N/A | N/A |
|  | CS fibrin | 15 (14 to 16) | N/A | N/A |
| Model 1 | Standard Care | 15 (14 to 17) | 15 (14 to 17) | 15 (14 to 17) |
|  | CS fibrin | 14 (13 to 16) | 14 (13 to 16) | 14 (13 to 16) |
| Model 2 |  |  |  |  |
| Drain + | Standard Care | 13 (11 to 16) | 13 (10 to 16) | 13 (11 to 16) |
|  | CS fibrin | 14 (12 to 16) | 14 (12 to 16) | 14 (12 to 16) |
| Drain - | Standard Care | 16 (14 to 18) | 16 (14 to 18) | 16 (14 to 18) |
|  | CS fibrin | 14 (13 to 16) | 14 (12 to 16) | 14 (13 to 16) |
