# Supplementary material for: The effect of a fibrin sealant on knee function after total knee replacement surgery. Results from the FIRST trial. A multicenter randomized controlled trial
Source: PLoS One. 2018 Jul 25;13(7):e0200804. doi: 10.1371/journal.pone.0200804 (PMC6059473; doi:10.1371/journal.pone.0200804)
Supplement: S1 Fig — (DOCX) [file pone.0200804.s005.docx]

**S1 Fig.
KOOS subscales preoperative and through follow-up as an out-come profile for Standard Care vs. CryoSeal group.**

**
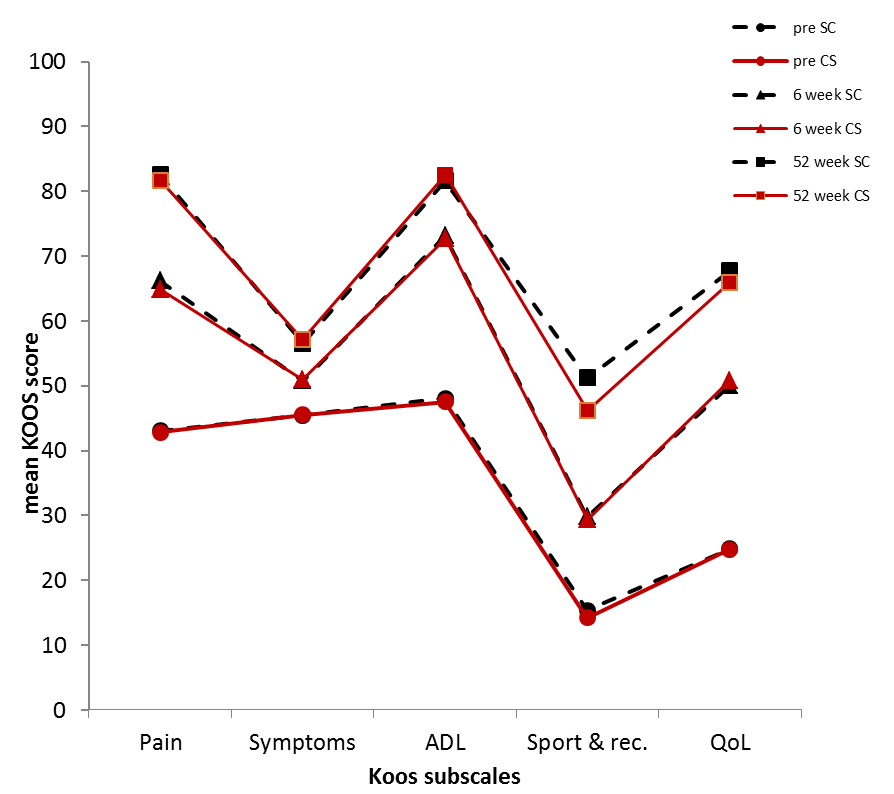
**

**SC= Standard Care; CS= CryoSeal; ADL = activity and daily life; QoL = quality of life**
