# Supplementary material for: The effect of a fibrin sealant on knee function after total knee replacement surgery. Results from the FIRST trial. A multicenter randomized controlled trial
Source: PLoS One. 2018 Jul 25;13(7):e0200804. doi: 10.1371/journal.pone.0200804 (PMC6059473; doi:10.1371/journal.pone.0200804)
Supplement: S1 File — (DOCX) [file pone.0200804.s006.docx]

**S1 File.**
**Transfusion protocol**

Patients younger than 60 years
Within 4 hours after surgery More than 4 hours after surgery
Hb ≥ 4.0 mmol / l = 0 packed cell Hb ≥ 4.0 mmol / l = 0 packed cell
3.0 - < 4.0 = 1 packed cell 3.5 - < 4.0 = 1 packed cell
< 3.0 = 2 packed cells < 3.5 = 2 packed cells

Patients older than 60 years
Within 4 hours after surgery More than 4 hours after surgery
Hb ≥ 4.5 mmol / l = 0 packed cell Hb ≥ 5.0 mmol / l = 0 packed cell
4.0 - < 4.5 = 1 packed cell 4.5 - < 5.0 = 1 packed cell
 < 4.0 = 2 packed cells < 4.5 = 2 packed cells

Patients with increased risk (because of co-morbidity)
Within 4 hours after surgery More than 4 hours after surgery
Hb ≥ 5.5 mmol / l = 0 packed cell Hb ≥ 6.0 mmol / l = 0 packed cell
5.0 - < 5.5 = 1 packed cell 5.5 - < 6.0 = 1 packed cell
4.5 - < 5.0 = 2 packed cells 5.0 - < 5.5 = 2 packed cells
< 4.5 = 3 packed cells < 5.0 = 3 packed cells

In all cases these are transfusion guidelines, of which the clinical presentation of the
patient is of greater importance to which transfusion policy is followed.
