# Supplementary material for: The effect of a fibrin sealant on knee function after total knee replacement surgery. Results from the FIRST trial. A multicenter randomized controlled trial
Source: PLoS One. 2018 Jul 25;13(7):e0200804. doi: 10.1371/journal.pone.0200804 (PMC6059473; doi:10.1371/journal.pone.0200804)
Supplement: S2 File — (DOC) [file pone.0200804.s007.doc]

**‘The use of Fibrin Sealant In**

**total knee Replacement Surgery Trial.’**

**FIRST-study**

A prospective, randomised multi center study

ORTHOPEDIC DEPARTMENTS

- Leiden University Medical Center, Leiden
- Bronovo Hospital, The Hague
- ‘t Groene Hart Hospital, Gouda
- Haga Hospital, The Hague
- Spaarne Hospital, Haarlem
- Amphia Hospital, Breda

**‘THE USE OF FIBRIN SEALANT IN KNEE REPLACEMENT SURGERY.’**

| **Protocol ID** | CryoSeal |
| --- | --- |
| **Short title** | CryoSeal in primary TKR (FIRST study) |
| **Title** | ‘The use of fibrin sealant in knee replacement surgery’ |
| **Version** | Version 4.1 |
| **METC nr.** | P10.115 |
| **Date** | 15-05-2013 |
| **Coordinating investigator/** | Dr J.A van Hilten  research investigator/coordinator |
| **Principal investigator(s) (hoofdonderzoeker/ uitvoerder):** | Prof. dr. R.G.H.H. Nelissen |
| **Writing/ Steering committee** | Prof. dr. R.G.H.H. Nelissen, LUMC  Dr. J.A. van Hilten, Sanquin Research  Dr J.G. van der Bom, Sanquin Research  Dr. E.W. van Zwet, Medical Statistics LUMC  Dr. H.M.J. van der Linden, LUMC |

**PROTOCOL SIGNATURE SHEET**

| **Name** | **Signature** | **Date** |
| --- | --- | --- |
| **Coordinating investigator/contact person** |  |  |
| **Principal Investigator:** |  |  |

TABLE OF CONTENTS

LIST OF ABBREVIATIONS AND RELEVANT DEFINITIONS [6](#__RefHeading___Toc250726688)

SUMMARY 7

1. INTRODUCTION AND RATIONALE 8

2. OBJECTIVES 10

3. STUDY DESIGN 11

4. STUDY POPULATION 13

4.1 Population (base) 13

4.2 Inclusion criteria 13

4.3 Exclusion criteria 13

4.4 Sample size calculation 13

5. TREATMENT OF SUBJECTS 14

5.1 Investigational product/treatment 14

5.2 Summary of known and potential risks and benefits 15

6. METHODS 16

6.1 Study parameters and endpoints 16

6.2 Randomisation, blinding and treatment allocation 17

6.3 Study procedures 18

6.4 Withdrawal of individual subjects 18

6.5 Replacement of individual subjects after withdrawal 18

7. SAFETY REPORTING 19

7.1 Section 10 WMO event [19](#__RefHeading___Toc250726708)

7.2 Adverse and serious adverse events [19](#__RefHeading___Toc250726709)

7.3 Follow-up of adverse events [19](#__RefHeading___Toc250726710)

8. STATISTICAL ANALYSIS 20

9. ETHICAL CONSIDERATIONS 22

9.1 Regulation statement 22

9.2 Recruitment and consent 22

9.3 Compensation for injury 22

9.4 Incentives 23

10. ADMINISTRATIVE ASPECTS AND PUBLICATION 23

10.1 Handling and storage of data and documents 23

10.2 Amendments [22](#__RefHeading___Toc250726719)

10.3 Annual progress report 23

10.4 End of study report 23

11. REFERENCES 24

APPENDIX 1: PROTOCOL RESTRICTIVE TRANSFUSION POLICY 26

APPENDIX 2: DEFINITIONS POSTOPERATIVE INFECTIONS 27

**Addendum:** analysis plan

# LIST OF ABBREVIATIONS AND RELEVANT DEFINITIONS

| **AE** | Adverse Event |
| --- | --- |
| **AR** | Adverse Reaction |
| **ASA** | American Society of Anaesthesiologists scale |
| **BMI** | Body Mass Index (weight in (kilogram)/ squire length (meters)) |
| **CA** | Competent Authority |
| **CCMO** | Central Committee on Research Involving Human Subjects |
| **CS** | CryoSeal |
| **CV** | Curriculum Vitae |
| **EU** | European Union |
| **EudraCT** | European drug regulatory affairs Clinical Trials GCP Good Clinical Practice |
| **F/E** | Flexion and Extension (knee function measured with goniometer) |
| **GMP** | Good Manufacturing Practice |
| **IB** | Investigator’s Brochure |
| **IC** | Informed Consent |
| **IMP** | Investigational Medicinal Product |
| **IMPD** | Investigational Medicinal Product Dossier |
| **INR** | International Normalized Ration (prothrombin time) |
| **METC** | Medical research ethics committee (MREC) (METC=Medisch Ethische Toetsing Commissie) |
| **MRC** | Medical Research Council scale; muscle power grading scale from 0-5 |
| **NSAID** | Non-steroidal anti-inflammatory drugs |
| **OR** | Operation Room |
| **(S)AE** | (Serious) Adverse Event |
| **Sponsor** | The sponsor is the party that commissions the organisation or performance of the research, for example a pharmaceutical company, academic hospital, scientific organisation or investigator. A party that provides funding for a study but does not commission it is not regarded as the sponsor, but referred to as a subsidising party. |
| **SUSAR** | Suspected Unexpected Serious Adverse Reaction  **TKA** Total Knee Replacement |
| **VAS** | Visual Analogue Scale |
| **WBP** | Personal Data Protection Act (WBP=Wet Bescherming Persoonsgevens) |
| **WMO** | Medical Research Involving Human Subjects Act (WMO=Wet Medisch-Wetenschappelijk Onderzoek met Mensen) |

# SUMMARY

**Rationale:** Fibrin Sealant is known to have some potential benefits for intraoperative use, especially in the acceleration of the coagulation cascade. Most studies with fibrin glue are focussing on the reduction of allogeneic blood transfusion and the possibilities to reduce costs. Firstly, fibrin sealant was made from patients’ blood plasma but the logistic problems made introduction in general hard. Now Sanquin has produced a CryoSeal (fibrin sealant) made of single-donor plasma which can be used during surgery. This application is already used in cardiothoracic surgery.

**Objective:** Is the application of CryoSeal (CS) in primary total knee replacement surgery beneficial for the patient with respect to a faster track and improved postoperative rehabilitation. The beneficial effect may be due to quicker functional knee rehabilitation, by pain reduction, better mobilisation and improved quality of life during the early postoperative period.

**Study design:** Multicenter, randomised controlled trial stratified by clinic.

**Study population:** Patients aged above 18 years scheduled for primary total knee replacement (TKA) because of osteoarthritis or rheumatic arthritis, meeting all the inclusion criteria and none of the exclusion criteria, are eligible for inclusion in the study.

**Intervention:** The intervention is the application of CryoSeal during surgery according to the standardised manner. The control group will receive standard care.

**Main study parameters/endpoints:** Primary endpoint: Difference in extension knee function 2 and 6 weeks after surgery. Secondary endpoints: postoperative complications, Pain score, knee function Flexion/Extension, Barthel score (day 3); Outpatient department scores (2 weeks, 6 weeks and 3 months, 1 year): Complications, Pain knee function, KSS, KOOS, SF-12, IPQ-K and EQ5D. A pre- and postoperative hand strength measurement will be performed since this might be a predictor of outcome after TKA. (Taekema 2010). Where possible, an actual physical activity measurement will take place to objectively analyse pre- and post operative acitivity level. (Groot, de 2008)

**Nature and extent of the burden and risks associated with participation benefit and group relatedness:** Given the origin of the CryoSeal product, i.e. donor plasma and in the absence of added bovine or chemical fibrinolysis inhibitors, no toxicity is to be expected by applying CryoSeal produced by the CS-1 (machine) / CP-3 (disposable) system, which was confirmed by pre and clinical research.

# 1. INTRODUCTION AND RATIONALE

Fibrin Sealant, also referred to as “fibrin glue” consists of two components, one component is thrombin and the other one is cryoprecipitate with fibrinogen. When both components are combined the last step of the coagulation cascade is started; the conversion of fibrinogen in fibrin. CryoSeal can be used at the end of the operation to seal the bare bony surfaces (i.e. bone surfaces not covered by the prosthesis), and synovial tissue with the aim to decrease or prevent large postoperative bleeding at the surgical field. The patient’s coagulation system is also capable to stop excessive bleeding, but this will probably take longer. CryoSeal only fastens this process because of the higher amount of growth factors; it imitates the last phase of the coagulation process. [Spotnitz, Am J Surg 2000]

Fibrin sealant finds its application in open cardiac surgery, hip- and knee surgery, liver surgery and plastic surgery.

Working mechanism

Haemostasis is achieved when there is a good balance between coagulation, the complement of this and the conversion of fibrinogen in fibrin under influence of thrombin. This coagulation cascade is known with the complex interaction of plasma proteins, blood cells, blood flow and viscosity and endothelia of the blood vessels.

After tissue damage, platelets are activated and stick to the blood vessel, changes of the vascular tone and aggregation is started. After activation platelets produce thromboxane A2, inositol-3-phosphate and diacylglycerol, who further regulate the activation of platelets and the release of Ca^2+^. Ca^2+^ is an important co-factor in the different coagulation stages.

Furthermore the outer membrane of the platelets is transformed and in this manner creating a good surface for coagulation, the production of thrombin and in lengthening prolonged the production of fibrin. [Mackie, biology of haemostasis and thrombosis, 1996]

The several investigations with the application of fibrin sealant or platelet rich plasma show contradictions in outcome. Levy et al. found positive results of fibrin sealant in the amount of blood loss during and after surgery in total knee replacements. [Levy, JBJS-Am 1999] The conflicting results are caused by the product that’s used outcome variables and study design. At the moment it is possible to make fibrin sealant as an autologous component out of patient’s blood plasma or as a single-donor product produced by the blood bank.

The advantage of autologous fibrin sealant is that the patient does not receive donor blood and that fibrinolysis reducers are not necessary. Disadvantages are mostly of logistic and economic nature. The general condition of the patient must be adequate; furthermore the blood is not always used. Most of all GMP circumstances are difficult to realise at the OR.

Everts et al. compared the different manufacturing options of platelet gel and concluded that the platelet amount can be increased five fold in comparison with whole blood. But the quality of the end product is strongly dependent on the basic product. [Everts, Growth Factors 2006]

Sanquin has started the production of fibrin glue, CryoSeal (CS), of allogene single donor plasma as alternative. The production is equal as autologous fibrin glue. Only the plasma that is used for the production is quarantine plasma that is Parvo B19-safe. The production is performed under regulated circumstances which are GMP proved. The CS is saved deep freeze and is always available, only has to be thaw.

CS is currently under study on reducing transfusion needs during coronary artery bypass surgery in 4 Dutch Thorax centres, the so called FIBER study. In this prospective randomised multi-centre trial of approximately 1500 patients the primary endpoints are amount of allogeneic blood transfusions and the duration of intensive care stay. The results are not yet available but the pilot study showed no side effects or CryoSeal related complications.

This study proposal may provide evidence based support for improved postoperative rehabilitation by using CS after total knee surgery and will contribute to optimal blood product management in orthopaedic surgery patients. The mechanism for this improved rehabilitation may by through reduction of the intra-articular bleeding, resulting in subsequent reducing postoperative pain and facilitating earlier postoperative functional improvement i.e. faster gain of knee flexion and knee extension. Sanquin’s centre for clinical research located at the Blood Bank Region Southwest approves designs and coordinates randomised controlled trials (RCT) together with principle investigators. Initiated from this collaboration, this project is a continuation of previously completed clinical research projects (TOMaat, Trigger & Drain studies) with the department of orthopaedic surgery of the LUMC (head: prof.dr. Rob G.H.H. Nelissen) and Sanquin researchers. The main objective of these studies was to define optimal transfusion management in orthopaedic surgery: Transfusie op Maat: ‘TOMaat study’. The infrastructure of the TOMaat trial can be used for the current question; on the clinical effectiveness, postoperative pain and early knee function of CS use in knee surgery.

# 2. OBJECTIVES

Is the application of CryoSeal (CS) by primary total knee replacement surgery beneficial for the patient with respect to a faster track and improved early (2 and 6 weeks) postoperative rehabilitation as determined by improved knee extension. The beneficial effect may be due to quicker rehabilitation (focus on knee function), by pain reduction, better mobilisation and improved quality of life (especially short term).

.

# 3. STUDY DESIGN

The study is a randomised, multi centre trial in orthopaedic surgery in patients who receive a primary total knee replacement. Patients will be randomised in two groups. The intervention group will be treated with CryoSeal per operative and the control group will receive standard care (no additional treatment during surgery). Patients will be stratified prior to randomisation in six strata occording to the the clinical site where the use of postoperative wound drain is the standard procedure. No consensus between the sites exists on the usage of the latter.

Patients and observers will be blinded for treatment until 3 months postoperative.

See Flow chart next page

**Baseline**

Medical History- KSS

Knee function (F/E)- KOOS

Pain score- EQ5D

Handstrength- SF-12

Barthel Index score- IPQ-K

Oxford Knee Score

**Stratification and randomisation**

Patient meeting in- and exclusion criteria and willing to participate in the clinical trial

**CryoSeal**

**Usual care**

**Surgery**

**With or without**

**application of CryoSeal**

**Hospitalisation**

- Clinical data
- Complications
- Pain score
- Knee function F/E
- Barthel score (day 3)

**Outpatient department (2 weeks, 6 weeks and 1 year)**

- Complications (all timepoints)
- Pain score (all timepoints)
- Knee function F/E (all timepoints)
- KSS (6 wks, 1 year)
- Oxford Knee Score (OKS) (2, 6 wks, 1 year)
- Barthel index (2, 6 wks and 1 year)
- KOOS (6 wks and 1 year)
- SF-12 (6 wks and 1 year)
- IPQ-K (6 wks and 1 year)
- EQ5D (2, 6 wks and 1 year)

# 4. STUDY POPULATION

## 4.1 Population (base)

Patients older than 18 years of age, undergoing primary total knee replacement surgery are included. Since total knee replacement surgery is a well defined standardised surgical procedure and CS application can be standardised on the amount and the well defined layers of femoral and tibial and patellar ‘if applicable’.

## 4.2 Inclusion criteria

- Patients who will undergo primary total knee replacement surgery for osteoarthritis or rheumatic arthritis
- Age, minimum of 18 years
- (Admission of the patient after informed consent)
- ASA classification I-III
- Capable of reading and answering the Dutch language

## Exclusion criteria

- Liver failure
- Congenital or acquired coagulation disorders
- Patients with known haemophilia or von Willibrand disease
- Patients with INR >2 (standard practice for operation)

## 4.4 Sample size calculation

We performed a sample size calculation for our primary outcome which is pre- to postoperative difference in knee extension angle at two weeks after surgery. We expect to find a difference between study arms of 10 degrees, which we also feel is clinically relevant. We have little prior data to base our calculations on, but we expect a standard deviation of 35 degrees in our primary outcome. We conclude that we need 250 patients in each arm to be able to detect the stated difference at significance level 4.9% with 90% power.

We have made a slight adjustment to the usual significance level of 5% because we plan to perform an interim analysis when a total of 250 patients have been included. At this time we will decide if the trial should be terminated early for efficacy or futility. If the trial continues, we will use the available data to estimate the standard deviation of the primary outcome. Based on this estimate, we will re-compute the sample size that is needed to achieve 90% power to detect a 10 degree difference between the study arms at level 5%. We will adjust the sample size accordingly. However, we will not include less than 400 patients or more than 600 patients.

# 5. TREATMENT OF SUBJECTS

## 5.1 Investigational product/treatment

ThermoGenesis (in the Netherlands represented by MDM) is a company that manufacturers a device for the production of fibrin sealant from single donor plasma, the CryoSeal Fibrin Sealant System (CS-1). Due to a low plasminogen concentration in the cryoprecipitate no fibrinolysis inhibitors are needed.

The following product developing activities are foreseen:

1. CS-maintenance longer than 2 years after production; 2. CS-Packing; 3. Cryo-poor plasma (re)use; 4. Development of custom-made (friendly use) disposables in collaboration with ThermoGenesis and MD-management.

Fibrin sealant will be produced and released from SBNO according to the operational production procedures. The production of fibrin sealant has been validated according the procedure VP-P.06.233. The quality criteria for fibrin sealant are: Cloth test: cloth formation < 10s; Qualitative FXIII test: no cloth resolving in 24h.

Preparation and application of the fibrin sealant

- The first procedures of fibrin sealant application was performed under supervision of experts from MDM. From these data it was decided to use two syringes of 5 ml each.
- Fibrin sealant will be used on the day of surgery in the operation room as follows:
- Thaw the fibrin sealant at 30-37°C, which takes about 10 minutes for the over wrapped fibrin sealant. Note: The thrombin and cryoprecipitate preparations from the cryoprecipitate system (CP-3) can be stored on ice for up to 4 hours, with 2 additional hours at 34-37°C or stored up to 6 hours at 34-37°C after which time they must be discarded.
- At the time of application as chosen by the surgeon, the fibrin sealant in the sterile field will be put as follows: Press on the locking bar inside the sterile over wrap to release the paired syringes from the plastic end connector (circulating nurse). Peel open the over wrap bag and present the paired syringes to another person (scrub nurse), who grasps only the end cap joining the two plungers and places the syringes into a tray in the sterile field. Repeat this for each set of paired syringes that is required for the patient.
- Assemble and load the FS Applicator with the fibrin sealant (scrub nurse).
- The circulating nurse peels the sterile over wrap of the Spray (ST-3) and drops it in the sterile field.
- FS Applicator tip is held facing upwards and the syringes lightly tapped to allow air bubbles to rise to the tip of the syringe (scrub nurse).
- The scrub nurse gently squeezes the plunger to expel any air bubbles into a sterile wipe, following this step a sterile luer lock stopcock, as available in the OR, will be placed on each of the Applicator tips transferred.
- Next the cryoprecipitate and thrombin preparations present inside the 3 cc paired syringes are prewarmed to 34-37°C during a 20 minute period. (To be used for pre-warming is the warning cabinet as present at the OR. The warming cabinet is to be set at a maximum temperature of 40°C. During pre-warning the CryoSeal pouches should NOT get in contact with any metal components inside the warming cabinet. Best is to place the CryoSeal (Cryolijm) pouches in a carton kidney-“bekkentje” available at anaesthesiology). After approximately 20 minutes the CryoSeal Fibrin Sealant, now pre-warmed to 34-37°C, is ready for use in the surgery field.
- The Fibrin Sealant will be applied to the surgical site. Two application techniques are possible. First, the kneeprothesis procedure is performed without the use a bloodbarrier until cementation of the prosthesis. In this technique the FS can be applied immediate after cementing the knee. A second technique is that the bloodless field is opened surgically temporarily to stop more extensive haemorrhaging via diathermy. Subsequently, pressure is re-applied followed by the application of the fibrin glue via the spray applicator on the surfaces of the dry wound bed. Furthermore the following steps are used: **A)** by holding the FS Applicator such that the tip is pointing toward the area targeted for haemostasis, **B)** positioning the tip approximately 7.5 – 8 cm from the surface of the target tissue, and **C)** pressing the plungers of the paired syringes while moving the hand in an “air brushing” back and forth type motion to create a homogeneous layer of fibrin sealant covering 15-17 square cm of target tissue per 1 mL of Fibrin Sealant. After waiting approximately 30 seconds, a second tip (ST-3) can be used to lay down a second layer of fibrin sealant on top of the first, following which a check for haemostasis should take. Vascular control is to be maintained throughout the application of the product.

## 5.2 Summary of known and potential risks and benefits

Given the origin of the CryoSeal™ product, i.e. donor plasma and in the absence of added bovine or chemical fibrinolysis inhibitors, no toxicity was / is to be expected by applying CryoSeal™ produced by the CS-1 (machine) / CP-3 (disposable) system, which was confirmed by pre and clinical research.

# 6. METHODS

## Study parameters and endpoints

Hypothesis

The hypothesis is that the application of CryoSeal (CS) during total knee replacement surgery is beneficial for the patient with respect to a faster track and improved postoperative rehabilitation. The beneficial effect may be due to quicker rehabilitation, by pain reduction, better mobilisation and quality of life (especially short time).

Primary endpoints

- postoperative knee function in extension difference as compared with preoperative extension function with goniometric at 2 and 6 weeks (in relation to secondary endpoints)

Secondary endpoints

- Grade flexion and extension difference after surgery
- Drain use or not
- Postoperative Hb/Ht
- Number of transfusions after surgery (according to appendix 1)
- Pain score
- Complications
- Total hospital stay and duration of rehabilitation
- Illness perception IPQ-K score
- Function improvement score (KSS, Barthel Index score, KOOS etc. see flowchart)
- Hand grip strength (measured with (Jamar) hand dynamometer)
- Activity Monitor

Study parameters

- Demographic data; gender, birth date (age), weight, length (BMI)
- Medical History; underlying diseases and home medication (including use of antibiotics prophylaxis and NSAIDs)
- Laboratory analysis; haemoglobin, haematocrit and blood view, CRP and erythrocyte sedimentation rate
- Operation details; anaesthesia, start- and end time of surgery, surgeon, type and time point placing and opening of drain, use of heparin, information about application of CryoSeal (if applicable), knee function after implantation of the implants
- Clinical data; number of transfusions (red cells, platelets and plasma), complications, removing drain, amount collected in drain, medication (including use of antibiotics and NSAIDs), discharge date
- Pain (score) at 24h post operative, and at 8:00 h each day until discharge from hospital.
- Rehabilitation; knee function during the first postoperative days, Barthel Index score and standard knee function scores (see Table 1).
- Handstrength measurement: is used as a proxy of muscle strength and measured by three maximal squeezes with both, the right and left hand applied at a hand held dynamometer (Jamar hand dynamometer, Sammons Preston Inc, Bolingbrook, IL)). Subjects are asked to stand up and hold the dynamometer in the hand with the arm parallel to the body without squeezing the arm against the body. The width of the handle is adjusted to the size of the hand to make sure that the middle phalanx rested on the inner handle. The participant is allowed to perform one test trial. After this, three trials are performed and the best score is taken for analysis. Handgrip strength is expressed in kilograms.

## 6.2 Randomisation, blinding and treatment allocation

Patients are included after signing the Informed Consent form. Patients will be stratified per clinic. Randomisation is done by web based computer application. Each centre will have its own username and password with a specific randomisation in blocks with a variable amount of patients. Block randomisation is used to get an equal distribution of patients in each treatment group for the interim and final analysis. Per randomisation patients characteristics like gender and date of birth are asked to prevent scrambling.

Each centre should randomise at least 50 patients to receive an equal distribution over the participating centres. Patient and assessors are blinded for the intervention by the patient. After 3 months follow-up, the treatment arm is communicated with the patient.

All patients are followed according to the intention-to-treat principle. Patients will be registered using a patient number, date of birth and study number and all other data will be coded by linkage to the study number.

An analysis plan will be enclosed as an addendum of this protocol 4.1 and before the study will be de-blinded.

**6.3 Study procedures**

| **Evaluation** | **Pre-op** | **OR (day 0)** | **Postoperative** | | | | | | |
| --- | --- | --- | --- | --- | --- | --- | --- | --- | --- |
|  |  |  | **Day 1** | **Day 2** | **Day 3** | **Discharge** | **2 wks** | **6 wks** | **1 year** |
| **Medical History** |  |  |  |  |  |  |  |  |  |
| **Operation details** |  |  |  |  |  |  |  |  |  |
| **Clinical data** |  |  |  |  |  |  |  |  |  |
| **Laboratory assessment** |  |  |  |  |  |  |  |  |  |
| **Complications** |  |  |  |  |  |  |  |  |  |
| **Pain score** |  |  |  |  |  |  |  |  |  |
| **Function (F/E)** |  |  |  |  |  |  |  |  |  |
| **Barthel score** |  |  |  |  |  |  |  |  |  |
| **KSS** |  |  |  |  |  |  |  |  |  |
| **KOOS** |  |  |  |  |  |  |  |  |  |
| **SF-12** |  |  |  |  |  |  |  |  |  |
| **Oxford Knee Score** |  |  |  |  |  |  |  |  |  |
| **IPQ-K** |  |  |  |  |  |  |  |  |  |
| **EQ5D** |  |  |  |  |  |  |  |  |  |

Table 1; parameters and time points of measuring

## 6.4 Withdrawal of individual subjects

Withdrawal is not to be expected, as standard implants are being used and follow-up is not different from routine follow-up of knee prosthesis in general. Subjects can leave the study at any time for any reason if they wish to do so without any consequences.

## 6.5 Replacement of individual subjects after withdrawal

Withdrawal is not to be expected, as standard implants are being used and follow-up is not different from routine follow-up of knee prosthesis in general. If patients withdraw from the study they will not be replaced.

Patients that are already randomised but operation is rescheduled because of any reason, subjects will stay in the study without changing randomisation.

# 7. SAFETY REPORTING

## 7.1 Section 10 WMO event

In accordance to section 10, subsection 1, of the WMO, the investigator will inform the subjects and the reviewing accredited METC if anything occurs, on the basis of which it appears that the disadvantages of participation may be significantly greater than was foreseen in the research proposal. The study will be suspended pending further review by the accredited METC, except insofar as suspension would jeopardise the subjects’ health. The investigator will take care that all subjects are kept informed.

## 7.2 Adverse and serious adverse events

Adverse events or adverse reactions are defined as any undesirable experience occurring to a subject during this randomised clinical trial. All adverse events reported spontaneously by the subject or observed by the investiga­tor or his staff will be recorded.

A serious adverse event (SAE) is any untoward medical occurrence or effect that at any dose results in death;

- is life threatening (at the time of the event);
- requires hospitalisation or prolongation of existing inpatients’ hospitalisation;
- results in persistent or significant disability or incapacity;

All serious adverse events will be reported to the accredited METC that approved the protocol, according to the requirements of that METC.

## 7.3 Follow-up of adverse events

All adverse events will be followed until they have abated, or until a stable situation has been reached. Depending on the event, follow up may require additional tests or medical procedures as indicated, and/or referral to the general physician or a medical specialist.

# 8. STATISTICAL ANALYSIS

Data will be analysed by the intention-to-treat principle. Demographic characteristics, variables from laboratory analysis and quantitative variables obtained from the clinical evaluation (Pain score, opioids consumption) will be reported as means, medians or percentages together with their standard deviations, as appropriate.

We will perform the following analysis of the primary endpoint. In all patients we test for a difference in mean pre- and post-operative extension angles between those with and without Cryoseal. Adjusting for pre-operative extension angles by analysis of covariance, we perform a two-sided test at level 0.049. In a secondary analysis, Fisher’s exact test will be used to determine the effect of use of fibrin sealant on adverse events. We will investigate whether the Cryoseal effect is modified by the use of a drain by adding drain use and the interaction between drain use and Cryoseal to the above analysis of covariance model. Also, effects within various other patients groups will be studied (e.g. gender, age, adipositas and rheumatic disorders)

Interim analysis and stopping rule:

We plan to perform a single interim analysis when a total of 250 patients have been included. At this time we will decide if the trial should be terminated early for efficacy. In all patients we test for a difference in mean pre- and post-operative extension angles between those with and without Cryoseal. Adjusting for pre-operative extension angles, we perform a two-sided test at level 0.005. If we are able to reject the null hypothesis, we terminate the trial and conclude efficacy of the treatment.

If the stopping rule is not met and the trial continues, we must set the level of significance of the final analysis to 0.049 to account for the fact that we performed an interim analysis. This is referred to as the O'Brien-Fleming rule.

Also, if the trial continues, we will use the available data to estimate the standard deviation of the primary outcome. Based on this estimate, we will re-compute the sample size that is needed to achieve 90% power to detect a 10 degree difference between the study arms at level 5%. We will adjust the sample size accordingly. However, we will not include less than 400 patients or more than 600 patients.

# 9. ETHICAL CONSIDERATIONS

## 9.1 Regulation statement

It is the responsibility of the investigator to obtain Medical Ethics Committee approval in writing, prior to the study start. A new approval for Medical Ethics Committee is required for any amendment or revision of the Clinical Investigation Plan affecting the intent and principles of the study or other ethical aspects. The investigator will maintain all correspondence with the Medical Ethics Committee.

## 9.2 Recruitment and consent

All patients have to review, understand, agree to, and personally sign and date the Informed Consent form prior to enrolment in the study. In case of subjects’ incompetence, a guardian or legal representative will have to assume the subjects role for the Informed Consent Procedure.

Informed Consent forms will be signed and dated by a medical doctor, or a Clinical Research Associate (CRA), authorised by the main investigator. The patient receives a copy of the Informed Consent form and the original form is filed in the study file.

## 9.3 Compensation for injury

The LUMC has a liability insurance which is in accordance with the legal requirements in The Netherlands (Article 7 WMO and the measure regarding Compulsory Insurance for Clinical Research in Humans of 23th June 2003). This insurance provides cover for damage to research subjects through injury or death caused by the study.

1. € 450.000 for death or injury for each subject who participates in the research;

2. € 3.500.000 for death or injury for all subjects who participates in the research;

3. € 5.000.000 for the total damage incurred by the organisation for all damage disclosed

by scientific research for the sponsor as ‘verrichter’ in the meaning of said Act in each year of insurance coverage.

The insurance applied to the damage that becomes apparent during the study or within for years after the end of the study.

De verzekeraar van het onderzoek is:

Name:  CentraMed

Adress:  Postbus 191, 2270 AD Voorburg

Tel.nr.: +31 (0)70-3017070

## 9.4 Incentives

Not applicable

# 10. ADMINISTRATIVE ASPECTS AND PUBLICATION

## 10.1 Handling and storage of data and documents

All personal data will be stored like the rest of the medical data according to the rules in our hospital. Only the investigation group has access to the source data of this study. Data will be kept until 15 years after finishing the study. When data will be used for publication they will never relate to individual traceable patients.

## 10.2 Amendments

Amendments are changes made to the research after an approval by the accredited METC has been given. All amendments will be notified to the METC that gave the approval.

A ‘substantial amendment’ is defined as an amendment to the terms of the METC application, or to the protocol or any other supporting documentation, that is likely to affect to a significant degree:

- the safety or physical or mental integrity of the subjects of the trial;
- the scientific value of the trial;
- the conduct or management of the trial; or
- the quality or safety of any intervention used in the trial.

All substantial amendments will be notified to the METC that gave a favourable opinion.

## 10.3 Annual progress report

The sponsor/investigator will submit a summary of the progress of the trial to the accredited METC once a year. Information will be provided on the date of inclusion of the first subject, numbers of subjects included and numbers of subjects that have completed the trial, serious adverse events/ serious adverse reactions, other problems and amendments.

## 10.4 End of study report

The investigator will notify the accredited METC at the end of the study. The end of the study is defined as the last patient’s last visit.

In case the study is ended prematurely, the investigator will also notify the accredited METC, including the reasons for the premature termination.

Within one year after the end of the study, the investigator will submit a final study report with the results of the study, including any publications/abstracts of the study, to the accredited METC.

# 11. REFERENCES

1. Milne AA. Clinical impact of fibrin sealants. Vox Sanquinis (2004) 87 (Suppl.2) S29-S30)

2. Ishimura M et al. Arthroscopic meniscal repair using fibrin glue. Arthroscopy 1997; 13:551-557

3. Crawford RW et al. Fibrin glue reduces blood loss in total hip arthroplasty. Hip Intl 1999; 9:127-132

4. Wang GJ et al. Use of fibrin sealant to reduce bloody drainage and haemoglobin loss after total knee arthroplasty. J of Bone and Joint Surgery 2001; 83:1503-1505

5. Wang GJ et al. Experience improves successful use of fibrin sealant in total knee arthroplasty: implications for surgical education. J Long Term Eff Med Implants 2003(5); 13: 385-397

6. Wang GJ et al. Fibrin sealant reduces perioperative blood loss in total hip replacement. J Long Term Eff Med Implants 2003; 13(5): 399-411

7. Mawatari M et al. Effectiveness of autologous fibrin tissue adhesive in reducing postoperative blood loss during total hip arthroplasty: a prospective randomized study of 100 cases. J Orthop Surg (Hong Kong). 2006 Aug;14(2):117-121

8. Kuhn DFM et al. The use of autologous fibrin glue to reduce peri-operative blood loss in total knee arthroplasty: results of a controlled study; Transfusion Alternatives in Transfusion Medicine 2005; 7.104, abstract p96.

9. Milic DJ et al. Prevention of pocket related complications with fibrin sealant in patients undergoing pacemaker implantation who are receiving anticoagulant treatment. Europace 2005, 7, 374-379

10. Zmora O, et al. Prospective, multicenter Evaluation of highly concentrated Fibrin Glue in the treatment of complex Crypotgenic Perianal Fistulas. Diseases of the Colon & Rectum 10.1007/s10350-005-0199-1

11. Spotnitz WD; Fibrin Sealant Tissue Adhesive Review and Update. Journal of Long-Term Effects of Medical Implants, 15(3)245-270 (2005)

12. Beierlein W, et al; Forty Years of Clinical Aprotinin Use: A Review of 124 Hypersensitivity Reactions. Ann Thorac Surg 2005; 79:741-748

13. Oswald A-M et al; Fatal intraoperative anaphylaxis related to aprotinin after local application of fibrin glue. Anesthesiology 2003; 99:521-523

14. Schlag MG et al; Convulsive seizures following subdural application of fibrin sealant containing tranexamic acid. Neurosurgery 2000; 47:1463-1467

15. Furtmuller R, et al; Tranexamic acid, a widely used anti-fibrinolytic agent causes convulsions. J Pharmacol Exp Ther 2002; 301:168-173

16. Pipan CM et al; Effects of antifibrinolytic agents on the life span of fibrin sealant. J. Surg Res 1992; 53:402-407

17. Beduschi R et al. Antifibrinolytic additives to fibrin glue for laparoscopic wound closure in urinary tract. J Endourol. 1999 May; 13(4):283-287

18. Marx G et al. Characterizing fibrin glue performance as modulated by heparin, aprotinin and factor XIII. J Lab Clin Med. 2002 Sep;140(3):152-160

19. Krishnan LK et al. Fibrinolysis inhibitors adversely affect remodelling of tissues sealed with fibrin glue. Biomaterials 2003 24; 321-327

20. Beierlein W et al. Forty Years of Clinical Aprotinin Use: A review of 124 hypersensitivity reactions. Ann.Thorac Surg 2005; 79:741-748

21. So-Osman, C et al. Efficacy, safety and user-friendliness of two devices for postoperative autologous shed red blood cell re-infusion in elective orthopaedic surgery patients: a randomised pilot study. Transfusion Medicine 2006; 16: 321-328

22. Taekema DG et al. Handgrip strength as a predictor of functional, psychological and social health. A prospective population-based study among the oldest old. Age and Ageing 2010; 39; 331-337

23. Groot de IB et al. Small increase of actual physical activtiy 6 months after total hip or knee arthroplasty. Clin Orthop Relat Res 2008; 466; 2201-2208

# APPENDIX 1: PROTOCOL RESTRICTIVE TRANSFUSION POLICY

Patients younger than 60 years

Within 4 hours after surgery More than 4 hours after surgery

Hb ≥ 4,0 mmol / l = 0 packed cell Hb ≥ 4,0 mmol / l = 0 packed cell

3,0 - < 4,0 = 1 packed cell 3,5 - < 4,0 = 1 packed cell

< 3,0 = 2 packed cells < 3,5 = 2 packed cells

Patients older than 60 years

Within 4 hours after surgery More than 4 hours after surgery

Hb ≥ 4,5 mmol / l = 0 packed cell Hb ≥ 5,0 mmol / l = 0 packed cell

4,0 - < 4,5 = 1 packed cell 4,5 - < 5,0 = 1 packed cell

< 4,0 = 2 packed cells < 4,5 = 2 packed cells

Patients with increased risk (because of co-morbidity)

Within 4 hours after surgery More than 4 hours after surgery

Hb ≥ 5,5 mmol / l = 0 packed cell Hb ≥ 6,0 mmol / l = 0 packed cell

5,0 - < 5,5 = 1 packed cell 5,5 - < 6,0 = 1 packed cell

4,5 - < 5,0 = 2 packed cells 5,0 - < 5,5 = 2 packed cells

< 4,5 = 3 packed cells < 5,0 = 3 packed cells

**In all cases these are transfusion guidelines, of which the clinical presentation of the patient is of greater importance to which transfusion policy is followed.**

# APPENDIX 2: DEFINITIONS POSTOPERATIVE INFECTIONS

1: Symptomatic urinary tract infections (UWI):

a) Positive urine culture with at least 1 clinical symptom of UWI *, or

b) At least 2 symptoms of UWI * with or

1) Urine positive in Gram-staining, or

2) Urine positive for leucocytes, or

3) Urine dipstick for nitrate or WBC esterase positive, or

4) Diagnosis UWI by clinician, or

5) Specific UWI therapy started by clinician

2: Asymptomatic UWI:

a) Positive urine culture with stay catheter or

b) Two positive urine cultures

3. Wound infections:

a) Purulent wound fluid, or

b) Positive wound culture, or

c) Diagnosis wound infection by clinician ^**^, or

d) At least one symptom of wound infection ^#^ with or

1) Conscious open wound made by the clinician, or

2) Spontaneous dehiscence of the wound

4: Pneumonia:

a) Crepitating or damping by percussion and

1) Positive blood-, bronchi- or bronchus biopsy culture with known respiratory pathogens, or

2) New purulent sputum or change in sputum appearance

b) X- thorax conforming pneumonia and

1) Positive cultures as above, or

2) Changes in sputum as above

5: Bacteraemia:

Positive blood culture with fever

* Symptoms of UWI: frequent free urge; dysurie; suprapubic pain; fever

# Symptoms of wound infection: pain; sensitive, local swelling, local warm and fever

** Referentie: W.J.Gaine et al., JBJS 2000; 82(4): 561-565

Addendum of FIRST protocol vs 4.1

dd 15 May 2013.

**Analysis plan**

The study database will be closed and subsequently be de-blinded more than six weeks after the last surgery procedure of the FIRST-patients. This analysis plan will be registered at the Dutch trial register (NTR) and the ethics committee will be informed about protocol 4.1 before execution of the plan using the data from the closed database. The primary analysis will be performed as stated in the protocol. Subsequently, a for three variables stratified subgroup analyses of the primary end points will be performed. This will be folllowed by a pre-specified subgroup analyses using 19 variables for hypothesis screening.

Statistical analyses

In SPSS, for each subgroup the odds ratio (OR under randomized controlled conditions) or relative risk (by univariate- and multivariate analysis (MVA)) with the corresponding 95% confidence interval (CI) will be estimated for CryoSeal. Although subgroups of the study population are predominately formed based on confounding factors, we will investigate the stability of the effect estimates over different levels of the confounding factors by correction in the MVA for those confounders. When a preoperative variable is significantly associated with CrySeal use we will address this observation, however we continue the primary and secondary analysis using the pre-specified subgroups.

Confounding and effect modification

Normally the problem of confounding by indication is virtually absent in the study of side effects of medical interventions. In case of CryoSeal use however, the subgroup analyses does not correct for the possibility of effect modification. Effect modification could arise since patients when for example wound drains were placed and could change the susceptibility of the patients for effects by CryoSeal.

Interpretation of pre-specified subgroup analyses and “multiple testing”

The stratified subgroup analyses will be made with each pre-specified subgroups. There will be no correction for multiple testing because all subgroups are defined by variables which are known to have underlying associations with the primary endpoints of the study. All subgroups were specified before the data are available. The implications of the outcomes will be discussed in relation to all other associated estimates observe in all subgroups.

The Plan of pre-specified subgroup analyses

Subgroup analyses:

After subsequent primary and secondary analysis of the effect of CryoSeal using the primary and secondary endpoints as presented in Protocol 4.1 of the total RCT dataset, according to intention to treat (ITT) and according to treatment (ATT), also pre-specified subgroup analyses of the putative effects on the primary and secondary endpoints (estimated odds ratio (OR) and 95% interval between both study-arms) of CryoSeal will be performed.

1. The following hypotheses will be tested using three for the variables stratified subgroups:

1. Catheter use and no catheter use
2. NSAID-users and no NSAID-users
3. Age > 70 and ≤ 70 years, or categorized in 3 equal sized samples of age (for example: <60, 60-70, >70)

The corresponding hypotheses are:

1. Catheter can disturb the CS-sealing process of the wound and therefore decrease the postoperative knee ROM and mobility effect of CS

2. NSAID-use are prescribed to reduce pain perception and as anti-coagulation medication, which decrease postoperative knee ROM and mobility effect of CS

3. Age is associated with increased co-morbidity resulting in reduced wound healing, which increase postoperative knee ROM and mobility effect of CS in patients > 60 years

2. Separated in the CryoSeal arm and in the non-CryoSeal patients the following 19 pre-specified subgroups will be selected for MVA to identify whether these factors are important or not in the CS-effect. Subsequently, using the total study cohort de following subgroups are analysed by dichotomic (or continuous) comparison (Y/N) between CryoSeal use or not:

Combined subgroups using 4 variables:

1. Catheter use and no catheter use
2. NSAID-users and no NSAID-users
3. Age > 70 and ≤ 70 years, or categorized in 3 equal sized samples of age (for example: <60, 60-70, >70)
4. Women and men

Single subgroups using each variable (or not):

1. Peroperative Hb ≤ 6 mmol/L and > 6 mmol/L
2. Transfused and non transfused patients
3. Prednison-users and no Prednison users
4. Cemented and no cemented protheses
5. “Bloedleegte” surgery and no “bloedleegte” surgery
6. If catheter use: Bellovac and no low-vac catheter use
7. If transfused: trigger of perioperative RBC use at Hb ≤ 6 mmol/L and > 6 mmol/L
8. If transfused: Age> 60 and age ≤ 60 with trigger of perioperative RBC use at Hb ≤ 6 mmol/L
9. Surgery time > 2 hr and < 2 hr

Stepwize variables:

1. When drain used: Wound salvage production or not (Y/N)
2. When salvage production, reinfusion or not

Continuous variable:

1. Hospital stay in days
2. Years of age

Categorized variables

1. Hospital stay: <3; 3-5; 6-8; >8 days
2. Age: >70; 70-60; <60 years of age

The subgroup analyses above are explorative for hypotheses screening.

Extra secondary analyses: Observational analyses of the RCT dataset:

When no correlation between the use of CryoSeal and the pre-, peri- and postoperative variables can be demonstrated and the above estimates are homogenous over the study arms, they will be pooled over the strata before further subgroup analysis; univariate followed by subsequent multivariate analysis will be performed.

The following research questions will be investigated by post-hoc analyses:

Preoperative variables:

1. Is there a correlation between preoperative hand grip strength and postoperative complications after > 1 year?
2. Are there associations between pre-op outcomes of the questionnaires and post-op functional/QoL/complications?
3. Is there a correlation between Range of Motion (ROM) and Oxford Knee Score outcomes?
4. Is there a correlation between preoperative NSAID (incl. Asperin) use and primary and secondary outcomes?
5. Is there a correlation between preoperative Prednison use and primary and secondary outcomes?

Perioperative variables:

1. Is there difference in post-op outcomes between LPS cemented vs cementless (Haga vs Spaarne) knee protheses
2. Is there difference in post-op outcomes (incl. hospital stay, body temp. (if applicable)) or -complications (incl. transfusion reactions) in transfused and non-transfused patients, or patients transfused at different indications/-triggers, and using different type of transfusion products
3. Are there more transfusion reactions in women?
4. Is there a difference in primary and secondary outcome between surgery type (“onder bloedleegte” or not)
5. Is there a difference in mean hospital stay and EPO use (if applicable)
6. Is there a correlation between wound catheter (incl. autologous reinfusion, if applicable) use vs no use and postoperative complications (bleeding, body temp).
7. Is there a correlation between perioperative wound drain reinfusion vs no reinfusion (if applicable) and postoperative complications
8. Is there a correlation between surgery time and postoperative outcomes of the questionnaires
9. Blood losses associated with type of catheter used
10. Is there a correlation between perioperative transfusion trigger (anaemia), age and length of hospital stay

Postoperative variables:

1. Is there a difference in postoperative complications (LoS, bleeding, pain, body temp.( if applicable)) with **reinfused autologous wound drain salvage** and no reinfused drain fluid after wound catheter use,
2. Costs calculation (hospital stay) of **drain use (all, incl. no autologous reinfusion) vs no drainage**, low-vac vs Bellovac vs no drainage (only Bronovo uses Bellovac, n=73),
3. postoperative QoL outcomes and drain use (all, incl. no autologous reinfusion)

List of postoperative outcomes (after 6 wk and 1 jaar)
- VG
- Medication
- Hand power
- BMI
- Barthel
- Oxford Knee Score
- KOOS
- SF12
- IPQ-K
- EQ5D
- Knee Society Score

Postoperative outcomes at hospital stay
-Postop complications, such as infections (deep and superficial wound infection, lung and urinary track infections),

-ROM and pain scores

-Postoperative bleeding

-Total hospital stay
-After two weeks: ROM, complicaties, pijnscore, Barthel, EQ5D, Oxford Knee

-Transfusion use: RBC transfusions, indications, triggers or type transfusion products
-Surgery time

Leiden, 15 May 2013
