# Supplementary material for: The effect of a fibrin sealant on knee function after total knee replacement surgery. Results from the FIRST trial. A multicenter randomized controlled trial
Source: PLoS One. 2018 Jul 25;13(7):e0200804. doi: 10.1371/journal.pone.0200804 (PMC6059473; doi:10.1371/journal.pone.0200804)
Supplement: S3 File — (DOC) [file pone.0200804.s008.doc]

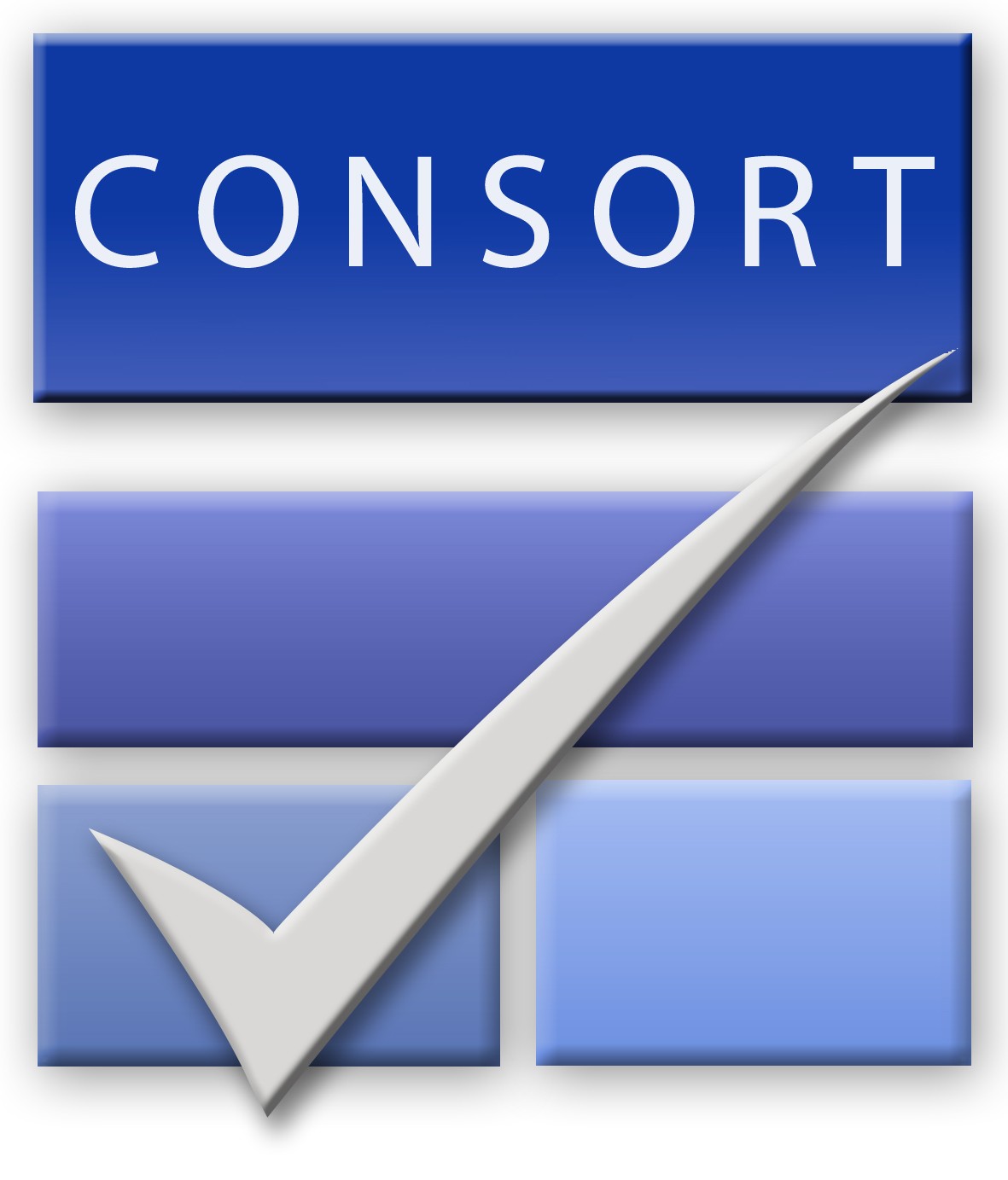
CONSORT 2010 checklist of information to include when reporting a randomised trial*

| Section/Topic | Item No | Checklist item | Reported on page No |
| --- | --- | --- | --- |
| Title and abstract | | | |
|  | 1a | Identification as a randomised trial in the title | P1 Line 3 |
|  | 1b | Structured summary of trial design, methods, results, and conclusions (for specific guidance see CONSORT for abstracts) | P2 |
| Introduction | | | |
| Background and objectives | 2a | Scientific background and explanation of rationale | P3 |
|  | 2b | Specific objectives or hypotheses | P3 Line 67-71 |
| Methods | | | |
| Trial design | 3a | Description of trial design (such as parallel, factorial) including allocation ratio | P4 Line 73 |
|  | 3b | Important changes to methods after trial commencement (such as eligibility criteria), with reasons | N/A |
| Participants | 4a | Eligibility criteria for participants | P4 Line 79-84 |
|  | 4b | Settings and locations where the data were collected | P6 Line 125-129 |
| Interventions | 5 | The interventions for each group with sufficient details to allow replication, including how and when they were actually administered | P4, Line 91-96, P5 Line 99-119 |
| Outcomes | 6a | Completely defined pre-specified primary and secondary outcome measures, including how and when they were assessed | P6, Line 130-138 |
|  | 6b | Any changes to trial outcomes after the trial commenced, with reasons | P6/7 Line 148-158 |
| Sample size | 7a | How sample size was determined | P6 Line 140-146 |
|  | 7b | When applicable, explanation of any interim analyses and stopping guidelines | P6/7 Line 147-158 |
| Randomisation: |  |  |  |
| Sequence generation | 8a | Method used to generate the random allocation sequence | P4, Line 85-88 |
|  | 8b | Type of randomisation; details of any restriction (such as blocking and block size) | P4, Line 85-88 |
| Allocation concealment mechanism | 9 | Mechanism used to implement the random allocation sequence (such as sequentially numbered containers), describing any steps taken to conceal the sequence until interventions were assigned | P4, Line 85-88 |
| Implementation | 10 | Who generated the random allocation sequence, who enrolled participants, and who assigned participants to interventions | Line 85-89; 108-111; 125-127 |
| Blinding | 11a | If done, who was blinded after assignment to interventions (for example, participants, care providers, those assessing outcomes) and how | Line 85-89; 108-111; 125-127 |
|  | 11b | If relevant, description of the similarity of interventions | N/A |
| Statistical methods | 12a | Statistical methods used to compare groups for primary and secondary outcomes | P7-8, Line 160-186 |
|  | 12b | Methods for additional analyses, such as subgroup analyses and adjusted analyses | Line 177-186 |
| Results | | | |
| Participant flow (a diagram is strongly recommended) | 13a | For each group, the numbers of participants who were randomly assigned, received intended treatment, and were analysed for the primary outcome | Figure 1 P20 |
|  | 13b | For each group, losses and exclusions after randomisation, together with reasons | P9 Line 189-196; P20 |
| Recruitment | 14a | Dates defining the periods of recruitment and follow-up | P9 Line 189 |
|  | 14b | Why the trial ended or was stopped | Line 155-156 |
| Baseline data | 15 | A table showing baseline demographic and clinical characteristics for each group | P18, table 1 |
| Numbers analysed | 16 | For each group, number of participants (denominator) included in each analysis and whether the analysis was by original assigned groups | P18 table 1 |
| Outcomes and estimation | 17a | For each primary and secondary outcome, results for each group, and the estimated effect size and its precision (such as 95% confidence interval) | Tables |
|  | 17b | For binary outcomes, presentation of both absolute and relative effect sizes is recommended | N/A |
| Ancillary analyses | 18 | Results of any other analyses performed, including subgroup analyses and adjusted analyses, distinguishing pre-specified from exploratory |  |
| Harms | 19 | All important harms or unintended effects in each group (for specific guidance see CONSORT for harms) | P19, table |
| Discussion | | | |
| Limitations | 20 | Trial limitations, addressing sources of potential bias, imprecision, and, if relevant, multiplicity of analyses | P12-13, Line 275-284 |
| Generalisability | 21 | Generalisability (external validity, applicability) of the trial findings | Line 267-274 |
| Interpretation | 22 | Interpretation consistent with results, balancing benefits and harms, and considering other relevant evidence | P11-12 |
| Other information | | |  |
| Registration | 23 | Registration number and name of trial registry | Line 14-15 |
| Protocol | 24 | Where the full trial protocol can be accessed, if available | N/A |
| Funding | 25 | Sources of funding and other support (such as supply of drugs), role of funders | P15 |

*We strongly recommend reading this statement in conjunction with the CONSORT 2010 Explanation and Elaboration for important clarifications on all the items. If relevant, we also recommend reading CONSORT extensions for cluster randomised trials, non-inferiority and equivalence trials, non-pharmacological treatments, herbal interventions, and pragmatic trials. Additional extensions are forthcoming: for those and for up to date references relevant to this checklist, see [www.consort-statement.org](http://www.consort-statement.org/).
